# Urgent investment needed to reclaim lactation care and training to support Indigenous women, babies, families, and communities

**DOI:** 10.1093/heapro/daag075

**Published:** 2026-06-11

**Authors:** Georgia Stewart, Summer M Finlay, Michelle Kennedy, Jessica Bennett, Alana Gall, Juanita Sherwood, Catherine Chamberlain, Michelle Dickson, Nicole Turner, Kristy Williams, Melanie Briggs, Simone Sherriff

**Affiliations:** Poche Centre for Indigenous Health, Edward Ford Building, The University of Sydney, Camperdown, New South Wales 2050, Australia; University of Wollongong, Northfields Avenue, Wollongong, New South Wales 2522, Australia; University of Wollongong, Northfields Avenue, Wollongong, New South Wales 2522, Australia; University of Newcastle, University Drive, Callaghan, New South Wales 2308, Australia; University of Newcastle, University Drive, Callaghan, New South Wales 2308, Australia; National Centre for Naturopathic Medicine, Southern Cross University, Military Road, Lismore, New South Wales 2480, Australia; University of Technology Sydney, 15 Broadway, Ultimo, New South Wales 2007, Australia; Waminda Women’s Health and Wellbeing Aboriginal Corporation, 122 Kinghorn Street, Nowra, New South Wales 2541, Australia; Indigenous Health Equity Unit, Melbourne School of Population and Global Health, University of Melbourne, 207 Bouverie Street, Melbourne, Victoria 3010, Australia; Poche Centre for Indigenous Health, Edward Ford Building, The University of Sydney, Camperdown, New South Wales 2050, Australia; Aboriginal Health and Medical Research Council of New South Wales, Level 4, 280 Pitt Street, Sydney, New South Wales 2000, Australia; Riverina Medical and Dental Aboriginal Corporation, 271 Edward Street, Wagga Wagga, New South Wales 2650, Australia; Waminda Women’s Health and Wellbeing Aboriginal Corporation, 122 Kinghorn Street, Nowra, New South Wales 2541, Australia; Poche Centre for Indigenous Health, Edward Ford Building, The University of Sydney, Camperdown, New South Wales 2050, Australia

**Keywords:** Indigenous, breastfeeding, lactation, self-determination, cultural medicines

## Abstract

Indigenous women have been nurturing and sustaining our babies through breastfeeding for thousands of years as an important practice for health, wellbeing, community and culture. Breastfeeding is key to giving our babies the best start to life. For many Indigenous women in Western colonized countries breastfeeding and cultural practices have been disrupted by the ongoing impacts of colonization. In this article, we draw on our lived experiences as Indigenous women with breastfeeding knowledges and a review on the field of breastfeeding support. We highlight how cultural breastfeeding practices and cultural medicines, including community-governed use of plant medicines, ceremony, massage and other practices defined by each Community, sustain breastfeeding, maternal wellbeing and infant health, yet remain marginalized in mainstream lactation care. There is a need to reclaim the lactation support space to have care that is grounded in our ways of knowing, being and doing. Indigenous voices have been sidelined from the breastfeeding support and lactation training space due to Western centric knowledges and a colonial lens being privileged. Therefore, prioritizing and privileging the voices and knowledges of Indigenous women and amplifying their experiences into the lactation field will improve breastfeeding rates and health outcomes. We call for the urgent investment into lactation care that prioritizes Indigenous knowledge systems, including cultural medicines, to support Indigenous women through their breastfeeding journeys. This requires funding, resources, investment, and support to build an Indigenous lactation workforce and recognize Indigenous-led lactation training as legitimate and necessary ways of supporting Indigenous women and families to breastfeed.

Contribution to Health Promotion

- Prioritizes culturally responsive and aligned lactation care for Indigenous women.
- Calls for Indigenous-led lactation training and workforce development in community-controlled health services.
- Amplifies Indigenous knowledges, healing, and voices in breastfeeding education, research, and training.
- Encourages long-term investment to support sustained actions and improve health outcomes for Indigenous babies and communities.
- Reclamation of Indigenous lactation knowledges and practices through lived and living experiences of Indigenous elders and women.

## Introduction

**Figure 1:**
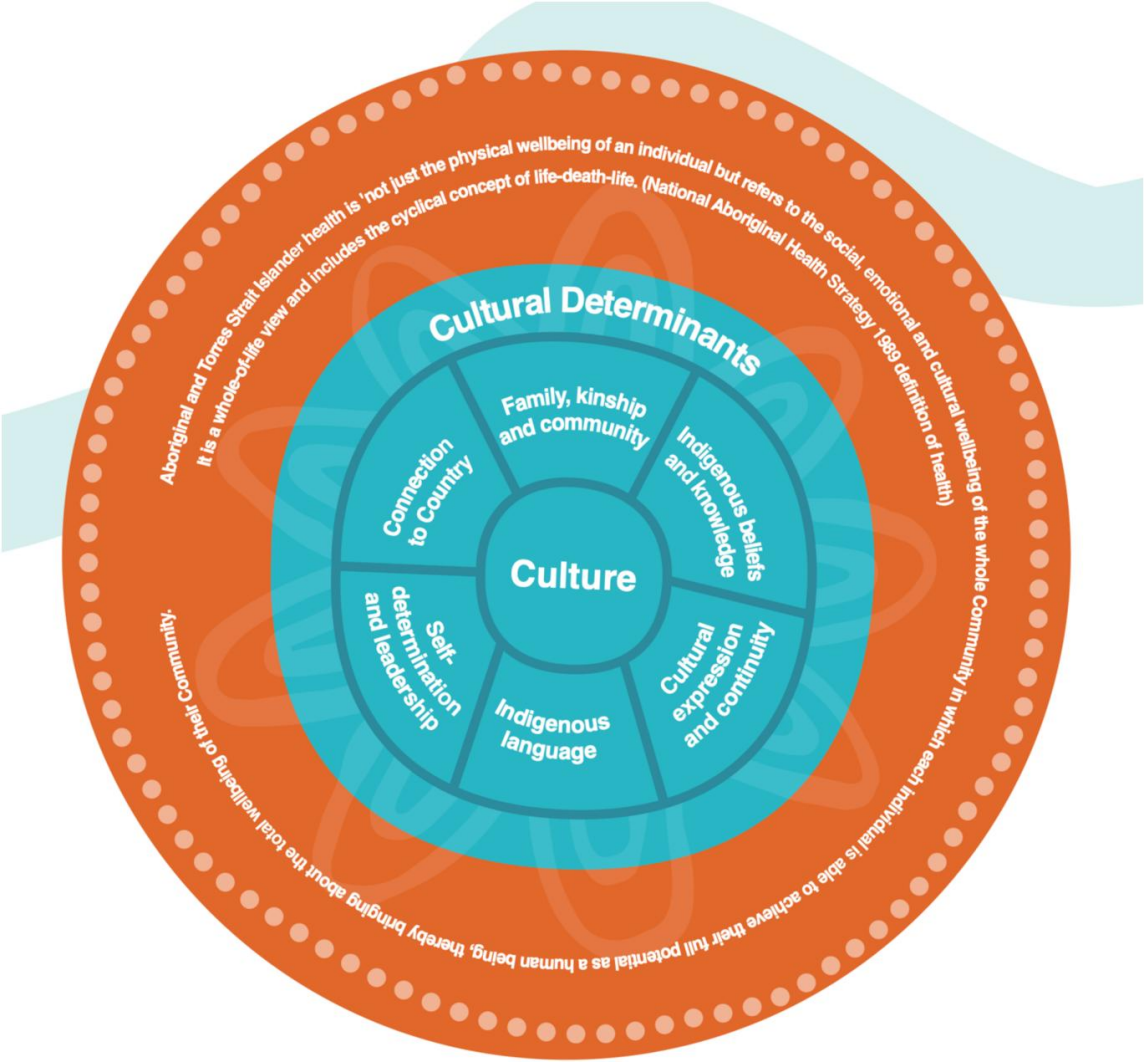
Cultural determinants of health domains identified by the Mayi Kuwayu study (Lowitja Institute 2020).

Indigenous women have been nurturing and sustaining our babies through breastfeeding for thousands of years as a first food sovereignty and as an important practice for health, wellbeing, community, and culture (Adams *et al*. 2018). Breastfeeding is foundational for food sovereignty in Indigenous communities, and empowers families to have control over infant feeding, wellbeing, health, and reclaiming cultural practices, through self-determination and autonomy (Cidro *et al*. 2015). Additionally, breastfeeding can mitigate the impacts of food insecurity and climate-driven environmental changes by providing a reliable source of nutrition that remains accessible through adverse weather events (Cidro *et al*. 2015). Despite this, breastfeeding is often left off the agenda including the National Inquiry into Food Security (2023) having little mention of breastfeeding (Parliament of Australia House of Representatives 2023). For Indigenous women and babies, breastfeeding is a sacred practice that is rooted in sovereignty and extends beyond nutrition (Gracey 2000). Breastfeeding is an intergenerational cultural practice that is learned through continuous observation, practice, and normalization. Indigenous breastfeeding is relational and a collective practice that was shared among women. These systems have been dismantled through colonization and colonial policies and systems (González-Grandón *et al*. 2026). Indigenous breastfeeding is embedded within cultural practices and shaped by cultural determinants of health (CDoH). It is also an act of resilience, resistance, and supports healing to break cycles of intergenerational trauma and reclaim Indigenous parenting practices. CDoH (Fig. 1) recognize culture as a protective factor for health and wellbeing for Indigenous peoples and communities (Lowitja Institute 2020). CDoH identify the benefits of culture and Country, rooted in Indigenous ways of knowing, being, and doing which can reduce health inequities (Lowitja Institute 2020). For many communities, breastfeeding is supported by cultural medicines, such as plant medicines, ceremony, massage, song, and story, that care for mothers, babies, and kin and are governed collectively by families, elders, and country (Gall *et al*. 2025). In this article, we draw on our lived experiences as Indigenous women with breastfeeding knowledges and a review on the field of lactation support.

## Positionality

This article is written by a collective of Indigenous women who come with our own diverse pregnancy, birth, and breastfeeding experiences in addition to our professional expertise in Indigenous health. We are mothers, grandmothers, aunties, and sisters whose perspectives are shaped by our roles in our communities, research, teaching, clinical practice, governance, and Aboriginal Community Controlled Organisations. Together, we form the Indigenous Lactation Collective in the country now known as Australia and we respectfully but strongly offer this commentary and a call to action. Through doing this we are upholding our sovereign rights to reclaim breastfeeding practices and calling on Australian governments to honour their commitment to funding Indigenous-led initiatives to support self-determination and better health and wellbeing outcomes.

## Indigenous Lactation Collective

The Indigenous Lactation Collective in Australia are calling for the development of lactation training programmes that are grounded in our ways of knowing, being, and doing. This will support the development of an Indigenous lactation workforce and ensure culturally responsive and aligned care is available to Indigenous women, children, and families. Although Australia has a National Breastfeeding Strategy, breastfeeding has not been elevated as a standalone Closing the Gap target, nor systematically reported across national Aboriginal and Torres Strait Islander health plans and strategies (COAG Health Council 2019, Australian Government 2021, Commonwealth of Australia 2025). Although Indigenous peoples have tens of thousands of years of breastfeeding knowledges, they continue to be excluded from the field of lactation training and breastfeeding support.

## Indigenous communities’ right to self-determine priorities for breastfeeding care

The upcoming 40th anniversary of the Ottawa Charter is an important reminder of the vision for ‘health for everyone’ underscored by the recognition that health is shaped by interconnected determinants (World Health Organization 1986, Thomas *et al*. 2025). Similarly, breastfeeding is also influenced by more complex determinants than individual behaviours (Sheehan *et al*. 2010, Moret-Tatay *et al*. 2025). For Indigenous women, these determinants intersect with ongoing impacts of colonization which has often silenced Indigenous peoples’ voices (Mitchell *et al*. 2023, Zheng *et al*. 2023). Ratima *et al*. (2019), assert that Indigenous knowledges and voices must be centred in health promotion to ‘promote planetary health, health equity, and sustainable development now and for future generations’ (p. 5). This assertion, coupled with the Ottawa Charter’s five action areas (building healthy public policy, creating supportive environments, strengthening community action, developing personal skills, and reorienting health services), are directly aligned with the vision to reclaim Indigenous knowledges and practices in breastfeeding and lactation training (World Health Organization 1986).

## The importance of breastfeeding

Breastfeeding is key to giving our babies the best start to life by providing short- and long-term health benefits for children and mothers. For babies, breastmilk supports healthy growth and development that adapts over time to meet the baby’s changing needs (Binns *et al*. 2016). Infants who are not breastfed are at an increased risk of respiratory and gastrointestinal infections, sudden infant death syndrome, and dental issues (Duijts *et al*. 2009, Khan *et al*. 2015). Later in life, not breastfeeding is linked to reduced cognitive outcomes and increased risks of obesity, asthma, and diabetes (Horta *et al*. 2015, Xue *et al*. 2021). For mothers, not breastfeeding increases the risk of postpartum bleeding and infection (Ji *et al*. 2018), and an increased risk of type 2 diabetes, cardiovascular disease, breast, and ovarian cancer (Ip *et al*. 2007). For these reasons, the World Health Organization recommends exclusive breastfeeding for the first 6 months of life (World Health Organization 2023). Following this period, it is recommended to introduce solid foods to complement breastfeeding, and breastfeeding should continue for a minimum of 2 years and beyond (World Health Organization 2023).

## Breastfeeding for Indigenous women

Despite the clear benefits of breastfeeding, there remains a scarcity of specific support for Indigenous women. In Australia, national data shows that 87% of Indigenous women initiate breastfeeding (Australian Government 2020). Despite these strong intentions, there is a sharp decline in breastfeeding rates among Indigenous children in Australia with 30% breastfed for at least 1 month to less than 6 months and 7% breastfed for 12 months (Australian Government 2020). Highlighting the need for culturally responsive breastfeeding support programmes for Indigenous women and families in Australia. The USA, New Zealand, and Canada Indigenous breastfeeding rates follow a similar pattern of high initiation rates before a decline at 6 months and beyond (Castro 2017, Public Health Agency of Canada 2022, Centers for Disease Control and Prevention 2023).

## Impacts of colonization and ongoing barriers for breastfeeding among Indigenous peoples

For many Indigenous women in countries with a similar colonial history, including Australia, breastfeeding and cultural practices and healing have been disrupted by the ongoing impacts of colonization (Ryan 2011, Monteith *et al*. 2024). Colonial events such as dispossession and forced removal from land have ignored Indigenous peoples’ sovereignty, and led to widespread trauma, population loss, and the disruption of some cultural systems, structures, and intergenerational transfer of knowledge (Monteith *et al*. 2024). Additionally, colonial policies including Assimilation, and the practice of the Stolen Generations, aimed to erase Indigenous cultures, languages, and familial structures (Human Rights and Equal Opportunity Commission 1997). These actions caused lasting and ongoing harm, severing cultural and community connections, and intergenerational knowledge transfer (Human Rights and Equal Opportunity Commission 1997).

Despite the end of formal child removal policies in the 1970s, Indigenous children in Australia are still disproportionately represented in the out of home care (OOHC) sector making up 44% of children in OOHC and being removed at a rate ten times those of non-Indigenous children (Liddle *et al*. 2023). Removal of infants has significant impacts on attachment between mothers and babies, parenting practices, and disrupts breastfeeding by challenging establishment or initiation (Peek *et al*. 2025). Similarly, in Canada, New Zealand, and the USA there is a clear pattern of the impacts of the events of colonization causing ongoing harm. Indigenous children are over-represented in the OOHC sector across Canada, New Zealand, and the USA (Oranga Tamariki (Ministry for Children) 2023, Puzzanchera *et al*. 2023, Hahmann *et al*. 2024). Indigenous mothers have the right to breastfeed their children and have access to care that is culturally safe, responsive, and aligned as stipulated in United Nations (UN) Declaration on the Rights of Indigenous Peoples and the UN Convention on the Rights of the Child (United Nations General Assembly 1989, United Nations 2007, United Nations Human Rights Office of the High Commissioner 2016). However, historical and ongoing racist and inequitable actions have removed this right for many Indigenous women around the world.

Additionally, the introduction of commercial milk formula, aggressively marketed as a substitute for human breastmilk, has negatively influenced breastfeeding rates contributing to a loss of breastfeeding knowledge (Rollins *et al*. 2023), and disrupted cultural feeding practices. Currently, structural pressures such as the rising cost of living crisis and limited access to affordable childcare often force mothers back to work earlier than intended (Springall *et al*. 2022). These past and present systems of injustices continue to impact Indigenous women’s ability to breastfeed.

## The medicalization of birthing practices

In addition, modern birthing practices continue to undermine Indigenous women’s ability to breastfeed in culturally supported ways. Including, barriers within the hospital system where there is a lack of culturally responsive policies and practices that support Indigenous women to be empowered to breastfeed in both maternal and neonatal care settings (Bennett *et al*. 2025, Hawke *et al*. 2025). Additionally, the growing medicalization of birthing practices is impacting on timely breastfeeding initiation, and the rate of caesarean births is projected to reach 29% of all births globally by 2030 (Betran *et al*. 2021). Among Indigenous women, a recent scoping review found that medicalization of childbirth for some Indigenous women resulted in a cultural and social disconnect (Sivertsen *et al*. 2025).

## International board-certified lactation consultant workforce

Internationally, the field of lactation support has been dominated by non-Indigenous health professionals and researchers, therefore these knowledges and voices are what make up most of the published literature. As with other health fields, this has led to a lack of Indigenous knowledges and perspectives to enrich the field of lactation support. Which in turn, has resulted in a lack of culturally responsive and aligned programmes and actions to support Indigenous breastfeeding (Zheng *et al*. 2023). Since the 1980s with the development of lactation consultants there has been a growing recognition of the important role lactation consultants play in many women’s breastfeeding journeys. Lactation consultants are specialist health professionals who support women to breastfeed their babies (Victoria State Government Department of Health 2024). Qualified lactation consultants are certified as International Board-Certified Lactation Consultant (IBCLC) meaning they have successfully completed the International Board of Lactation Consultant Examiners certification exam and met all minimum requirements (Victoria State Government Department of Health 2024).

## Indigenous lactation training and breastfeeding support

Currently, in Australia there are no Indigenous lactation training pathways, no data on the number of Indigenous lactation consultants or a central way for Indigenous women to locate an Indigenous lactation consultant for support. In 2019 the US Lactation Consultant Association surveyed the profession to obtain demographical data. Of the 346 responses collected, it was found that the overwhelming majority of IBCLCs and non-IBCLC lactation care providers identified as white women between the ages of 41–70 years (Harmon 2020). The data also showed that only 0.125% of IBCLCs in the USA are Indigenous highlighting the difficulty for Indigenous families to access an Indigenous IBCLC (Harmon 2020). Systemic barriers such as intergenerational poverty, education, and insurance restrictions are noted as hindering Indigenous peoples entering the lactation field (Sayers 2024). Anecdotally we know from Indigenous women who have undertaken the process to become an IBCLC it is difficult, costly, and not a culturally safe process, and therefore there are very few Indigenous lactation consultants in Australia. The stringent and narrow beliefs on what constitutes appropriate lactation care and support speaks to the field not always being an inclusive one for Indigenous peoples.

## Privileging Indigenous knowledges in lactation care

Importantly, Indigenous voices and knowledges have been sidelined from the lactation training space, therefore prioritizing and privileging the voices of Indigenous women and amplifying their experiences and knowledges into the lactation field will improve breastfeeding rates and health outcomes. The need for more Indigenous voices is apparent by the lack of published evidence that is led by Indigenous peoples, low numbers of Indigenous lactation consultants, and poor breastfeeding outcomes (Castro 2017, Australian Government 2020, Harmon 2020, Public Health Agency of Canada 2022, Centers for Disease Control and Prevention 2023, Mitchell *et al*. 2023, Monteith *et al*. 2024).

There is growing momentum within Indigenous communities across CANZUS (Canada, Australia, New Zealand, and the USA) to prioritize the breastfeeding and lactation field to include our collective ways of knowing, being, healing, and doing. Until recently, there were only Western models of lactation training available, leading to a critical gap in culturally safe and responsive lactation support for Indigenous women internationally. A movement led by two Indigenous women, Camie Goldhammer (Sisíthuŋwaŋ-Waȟpéthuŋwaŋ Oyáte) and Kimberely Moore-Salas (Naakaii Diné (Mexican) and Tsi’naajinii (Black Streak), who have developed (to our knowledge) the world’s first Indigenous lactation counsellor training programme (Sayers 2024). A programme designed by Indigenous women for Indigenous women and grounded in Indigenous ways of knowing, being, and doing. This programme has trained over 1200 Native and Indigenous women across the USA (including Hawaii and Alaska), Guam, and Canada, building a much-needed Indigenous lactation workforce in these countries (Sayers 2024). There is grey literature available on the importance of this training programme but no peer reviewed research to date. It is an example of successful Indigenous-led lactation training exemplifying what lactation care should look like. Additionally, the WHO’s Global Breastfeeding Scorecard 2023 identifies seven calls to action priorities which include ‘improve access to skilled breastfeeding counselling in healthcare facilities’, and ‘encourage networks that protect, promote and support breastfeeding’ (Castro 2017, World Health Organization 2023).

## Reclaiming Indigenous breastfeeding practices

The Western lactation model focuses on the mother-baby dyad which, while important, fails to incorporate or understand Indigenous worldviews of holistic lactation care that includes family, kin, community, cultural medicines, and Country. The current lactation care system is not working for Indigenous women, children, and families. Reclaiming Indigenous breastfeeding practices and knowledges will lead to better health outcomes for mums, babies, families, and communities. This reframes what breastfeeding supports look like for Indigenous women by providing safe spaces, intergenerational knowledge transfer, holistic care, and reconnection to cultural practices, healing, and protocols. We know the importance of cultural breastfeeding practices and cultural medicines, including the use of plant medicines, ceremony, massage, smoke, and other community-defined practices to support milk production, postbirth recovery, and infant health, and these need to be acknowledged and valued for their role in supporting breastfeeding. Medicalized birth practices, including high rates of caesareans and interventions, can interfere with getting the baby on the breast within 24 h window post birth, which is crucial to initiating and establishing breastfeeding, and the onset of milk transitioning (lactogenesis II) (Chen *et al*. 2026). Globally, the rate of caesarean births is projected to reach 29% of all births by 2030 (Betran *et al*. 2021). The Australian Institute of Health and Welfare reports in 2023 41% of mothers had a caesarean birth, increased from 32% in 2010 (Australian Institute of Health and Welfare 2026). Additionally, a recent scoping review found that medicalization of childbirth for some Indigenous women resulted in a cultural and social disconnect (Sivertsen *et al*. 2025).

As outlined in this paper, breastfeeding is food sovereignty and linked to good health and wellbeing, importantly including preventative health. Investing in the first days, weeks, months, and years of children’s breastfeeding can have positive ripple effects including supporting resilience and intergenerational healing among families. Therefore, we need to start at the beginning with investment into and prioritization of building an Indigenous lactation workforce to amplify Indigenous holistic wellness models of care for mothers and children.

To do this, there is the urgent need for the development of Indigenous lactation training that is recognized, acknowledged and valued as a legitimate way of supporting Indigenous women and families to breastfeed their children. This includes recognizing Indigenous-led lactation training, Aboriginal Community Controlled Health Organisations, and community Cultural Medicines practitioners as legitimate, expert providers of breastfeeding support. We are sovereign peoples and have a right to self-determine what lactation and breastfeeding care looks like, as stated in the United Nations Declaration on the Rights of Indigenous Peoples (United Nations 2007). There is a need to transform breastfeeding care for Indigenous peoples to have supports that are grounded in our ways of knowing, being, and doing which includes cultural medicines and practices to support birth and breastfeeding.

## Urgent investment is required into Indigenous lactation care

All women, children, and families have the right to access culturally safe and responsive lactation care. We call on the urgent investment into lactation care that prioritizes and embeds Indigenous knowledge systems to support Aboriginal and Torres Strait Islander women through their breastfeeding journeys. We call for the following actions:

1. Culturally responsive lactation training programmes.

The design and implementation of Indigenous lactation training programmes to be delivered through an Indigenous training organization

2. An Indigenous lactation workforce.

To ensure culturally grounded care we need the development of lactation skilled health professionals within the Aboriginal Community Controlled Health sector and mainstream health sector.

3. Transparent data collection processes.

To better understand the education pathways undertaken of people who have undertaken the International Board of Lactation Consultant (IBCLC) Exam to become an IBCLC. Additionally, the introduction of collecting data on the Indigenous status to inform the development of a central database of Indigenous IBCLCs or similarly trained Indigenous lactation workers for Indigenous women to access for support.

4. Prioritization of Indigenous knowledges and lived experiences.

To ensure breastfeeding policies, health care, programmes, and education are culturally responsive to the needs of Aboriginal and Torres Strait Islander women, families, and communities.

5. National data collection on Aboriginal and Torres Strait Islander breastfeeding initiation, duration, and experiences.

To collect data that is a priority for Aboriginal and Torres Strait Islander communities and that upholds Indigenous Data Sovereignty and Indigenous Data Governance, which will enable trends to be compared over time specific to our communities and data that can disaggregated that can be used by specific communities or geographical areas.

6. Recognition and resourcing of Cultural Medicines as foundational knowledge within Indigenous lactation care.

Investment to embed cultural medicines and knowledges into Indigenous breastfeeding training curricula, workforce roles, service models, research, and policy, which is governed by communities.

## Conclusion

Indigenous breastfeeding is a sacred, cultural practice underpinned by food sovereignty. Therefore, investing in Indigenous lactation care in Australia, and internationally, is a vital step towards intergenerational wellbeing, health justice, and cultural strengthening. Indigenous women have a right to access breastfeeding care that honours their knowledge systems and privileges their lived experience, while strengthening communities. The development and implementation of an Indigenous-led lactation training programme within Australia will have intergenerational impacts on the health and wellbeing of Aboriginal and Torres Strait Islander peoples and support in closing the gap. Central to this work is honouring cultural medicines and community-governed breastfeeding practices as core components of Indigenous lactation care, not optional add-ons, so that our babies, mothers, families, and communities can thrive.

## Data Availability

No new data were generated or analysed in support of this research.
